# Complete genome sequence of *Bacillus velezensis* strain Ag109, a biocontrol agent against plant-parasitic nematodes and *Sclerotinia sclerotiorum*

**DOI:** 10.1186/s12866-024-03282-9

**Published:** 2024-06-07

**Authors:** Silas Mian, Andressa Cristina Zamboni Machado, Rodrigo Thibes Hoshino, Mirela Mosela, Allan Yukio Higashi, Gabriel Danilo Shimizu, Gustavo Manoel Teixeira, Alison Fernando Nogueira, Renata Mussoi Giacomin, Luriam Aparecida Brandão Ribeiro, Alessandra Koltun, Rafael de Assis, Leandro Simões Azeredo Gonçalves

**Affiliations:** 1 Agronomy Department, Universidade Estadual de Londrina, Londrina, Paraná 86097-570 Brazil; 2 Nematology Department, Instituto Agronômico Do Paraná, Londrina, Paraná 86047-902 Brazil; 3 Microbiology Department, Universidade Estadual de Londrina, Londrina, Paraná 86097-570 Brazil; 4 Biology Department, Universidade Estadual Do Centro Oeste, Guarapuava, Paraná 85015-430 Brazil; 5 Center for Molecular Biology and Genetic Engineering, UNICAMP, Campinas, São Paulo, 13083-875 Brazil

**Keywords:** *Glycine max* (L.) Merr., Biological control, *Sclerotinia sclerotiorum*, *Meloidogyne javanica*, *Pratylenchus brachyurus*

## Abstract

Soybean is the main oilseed cultivated worldwide. Even though Brazil is the world’s largest producer and exporter of soybean, its production is severely limited by biotic factors. Soil borne diseases are the most damaging biotic stressors since they significantly reduce yield and are challenging to manage. In this context, the present study aimed to evaluate the potential of a bacterial strain (Ag109) as a biocontrol agent for different soil pathogens (nematodes and fungi) of soybean. In addition, the genome of Ag109 was wholly sequenced and genes related to secondary metabolite production and plant growth promotion were mined. Ag109 showed nematode control in soybean and controlled 69 and 45% of the populations of *Meloidogyne javanica* and *Pratylenchus brachyurus*, respectively. Regarding antifungal activity, these strains showed activity against *Macrophomia phaseolina*, *Rhizoctonia solani*, and *Sclerotinia sclerotiorum*. For *S. sclerotiorum*, this strain increased the number of healthy plants and root dry mass compared to the control (with inoculation). Based on the average nucleotide identity and digital DNA-DNA hybridization, this strain was identified as *Bacillus velezensis*. Diverse clusters of specific genes related to secondary metabolite biosynthesis and root growth promotion were identified, highlighting the potential of this strain to be used as a multifunctional microbial inoculant that acts as a biological control agent while promoting plant growth in soybean.

**Supplementary Information:**

The online version contains supplementary material available at 10.1186/s12866-024-03282-9.

## Introduction

Soybean (*Glycine max* (L.) Merr.) is considered the main oilseed cultivated in the world, with a total of 375 Mt of grain produced in 2023 (USDA – United States Department of Agriculture). Brazil produced approximately 142 Mt, with an increase of ~ 7.12 Mt year^−1^ and 91.41 kg ha year^−1^ during the last ten years (CONAB – Companhia Nacional de Abastecimento 2023). However, even with this favorable scenario, soybean yield and its expansion to new areas are severely affected by the restrictions imposed by biotic and abiotic factors [1–3].

Plant-parasitic nematodes are significant constraints to soybean production worldwide, accounting for a projected loss of $78 billion annually worldwide, with an average yield loss of 10–15% in soybean [4]. In Brazil, the main nematodes species that attack the soybean crop are root-knot nematodes (*Meloidogyne javanica* and *M. incognita*), cyst nematodes (*Heterodera glycines*), reniform nematodes (*Rotylenchulus reniformis*), and the root lesion nematode (*Pratylenchus brachyurus*) [5–7]. Other soilborne pathogens have also caused significant damage, such as white mold (*Sclerotinia sclerotiorum*) [8], root rot (*Rhizoctonia solani*) [9], charcoal rot (*Macrophomina phaseolina* (Tassi) Goid) [10], and sudden death syndrome (*Fusarium* sp.) [11]. It is estimated that approximately 30% of the Brazilian soybean area (~ 10 million ha) is infested with *S. sclerotiorum*, causing annual economic losses estimated at US$ 1.47 billion due to yield reduction and fungicide costs [12].

Controlling soil diseases poses significant challenges due to their diverse pathogens, resilient survival mechanisms, and dependence on complex soil ecology and environmental factors, necessitating multifaceted and adaptive management strategies for effective mitigation. Therefore, it is essential to carry out integrated management, which includes the rotation or succession of non-host crops, resistant or tolerant cultivars, and chemical and biological controls [13–15]. Biological control is increasingly favored for mitigating soil pathogens, owing to its effective suppression capabilities, minimal environmental footprint, and beneficial impacts on plants, including the induction of both local and systemic resistance as well as promotion of plant growth [16–18]. Furthermore, developing a commercial biological product costs 75 times less than developing a synthetic chemical [19].

In Brazil, there is a remarkably growth in the number of biological products for plant disease control, highlighting the genus *Bacillus* (https://agrofit.agricultura.gov.br/agrofit_cons/principal_agrofit_cons). *Bacillus* spp. represent a large group of gram-positive bacteria that belong to the phylum Firmicutes. It has been prominent in agriculture due to its diverse secondary metabolism and ability to produce various antagonist substances with different structures [20, 21]. For example, *B. velezensis* FZB42 has approximately 8% of its genome related to secondary metabolite production, including bacteriocins, antimicrobial peptides and lipopeptides, polyketides, and siderophores, which show plant growth-promoting properties [22, 23].

Considering the advances in biological control in recent years and the increased demand for these products in the market, this study aimed to evaluate the potential of the isolate *Bacillus velezensis* Ag109 as a biocontrol agent against soil pathogens in soybean, especially *M. javanica*, *P. brachyurus* e *S. sclerotiorum*. In addition, we sequenced the genome of Ag109 and mined the genes related to secondary metabolite synthesis and plant growth promotion.

## Material and methods

### Origin of the Ag109 strain

The bacterial strain Ag109 belongs to the AgBio company’s collection of microorganisms. The bacterium was isolated from the rhizospheric soil of maize harvested in Italva, Rio de Janeiro, Brazil. Its selection for this study was informed by earlier screenings among various bacterial isolates, which identified its antifungal activity and swarming ability.

### In vivo studies against *Meloidogyne javanica* and *Pratylenchus brachyurus* under greenhouse conditions

To evaluate the biocontrol activity of Ag 109 against *M. javanica* and *P. brachyurus*, two experiments were conducted for each pathogen at the Institute of Rural Development of Paraná (IDR-Paraná). For these experiments, seeds of the soybean cv. Bônus were used. The seeds were disinfected by immersion in 1% (v:v) sodium hypochlorite solution for 1 min. Then, they were subjected to triple washing by immersion in sterile distilled water and dried in a safety cabinet for four hours. Then, they were treated with biological and chemical products, as follows:

1. Treatment 1: Control
2. Treatment 2: Avicta 500 FS (active ingredient: Abamectin, applied at 50 mL/50 kg of seeds)
3. Treatment 3: Presence (*Bacillus subtilis* FMCH002 and *Bacillus licheniformis* FMCH001 applied at 75 g/50 kg of seeds)
4. Treatment 4: Votivo Prime (active ingredient: *Bacillus firmus* I-1582, applied at 200 mL/50 kg of seeds)
5. Treatment 5: Ag109 (active ingredient: *Bacillus velezensis* Ag109, applied at 200 mL/50 kg of seeds). The Ag109 was prepared from pure colonies suspended in saline solution (0.85% NaCl), and the turbidity was adjusted to 0.5 on the McFarland nephelometric scale. The inoculum was prepared from 30 µL of this bacterial suspension, which was transferred to a 125 mL Erlenmeyer flask containing 30 mL of LB broth and incubated at 28 °C for 24 h at 180 rpm in an orbital shaker incubator (Tecnal—TE 422, Brazil). For fermentation, an Erlenmeyer flask containing 400 mL of culture medium (20 g L^−1^ glucose, 5 g L^−1^ yeast extract, 5 g L^−1^ tryptone, 1 g L^−1^ monobasic potassium phosphate, 0.5 g L^−1^ dibasic potassium, 0.5 g L^−1^ magnesium sulfate, 0.5 g L^−1^ iron sulfate, 0.5 g L^−1^ calcium chloride, and 0.5 g L^−1^ sodium chloride) was inoculated with a 4 mL aliquot of the bacterial inoculum and incubated at 28 °C for 72 h at 180 rpm in an orbital shaker incubator (Tecnal—TE 422, Brazil). The fermentation of Ag109 was carried out as previously described, standardized for 1 × 10^9^ endospores mL^−1^.

The seeds were sown in 945-mL Styrofoam pots containing a mixture of sand and soil (81% sand, 14% clay, and 5% silt) previously sterilized (160° / 5 h). At planting, 3 g of Osmocote® (15% N, 9% P2O5, 12% K2O, 1% Mg, 2.3% S, 0.05% Cu, 0.45% Fe, 0.06% Mn, 0.02% Mo) was added per pot. Seven days after sowing (DAS), plants were inoculated with 1 mL of a suspension containing 1000 eggs of *M. javanica* or 500 eggs and juveniles/adults of *P. brachyurus*, which were extracted according to [24], without the use of sodium hypochlorite for *P. brachyurus*, and quantified in Peter’s slide under a light microscope. Sixty DAS, plant roots were washed free of soil in tap water, dried on absorbent paper, and weighed to obtain the fresh root mass (FRM). Subsequently, they were processed according to [24], and the eggs and juveniles were quantified in Peter’s slide under a light microscope. The nematode reproduction factor (RF = the number of nematodes in the sample/number of inoculated nematodes) and the number of nematodes per gram of roots (NGR) (the number of nematodes in the sample/ FRM) were determined. A completely randomized experimental design was adopted with ten replications.

### Activity of Ag109 against fungal soil-borne pathogens

#### Direct antagonism test by double culture

The fungi *Rhizoctonia solani*, *Sclerotinia sclerotiorum*, and *Macrophomina phaseolina* were provided by the GDM® company, Cambé, Paraná state, Brazil. To prepare the inoculum, the fungal strains were subcultured in Petri dishes containing potato dextrose agar (PDA) culture medium and incubated at 25 °C for two to five days, according to the fungus growth rate.

To prepare the bacterial inoculum, the Ag109 strain was spread over the agar surface of Luria Bertani (LB) culture medium in a Petri dish, incubated at 28 °C for 24 h, and subjected to a second cultivation under the same conditions. Three to five colonies were suspended in saline solution (0.85% NaCl), and the turbidity was adjusted to 0.5 on the McFarland nephelometric scale.

A 5 µL aliquot of the Ag109 inoculum was spread in a line using a bacteriological loop at 0.5 cm from the end of the Petri dishes containing PDA culture medium. Then, a 6 mm-diameter mycelial disc of the fungus (*R. solani*, *S. sclerotiorum*, or *M. phaseolina*) was inoculated at the opposite end of the bacterial inoculum. Plates with only the fungus were used as controls. The evaluation was carried out at different times, according to the growth rate of each fungus, 48 h for *R. solani* and 96 h for *S. sclerotiorum* and *M. phaseolina*. The mycelial growth of the fungi (in mm) was measured using a caliper, and the percentage of inhibition was calculated.

### Activity of the autoclaved fermentation broth and cell-free supernatant of Ag109 on the mycelial growth of *Sclerotinia sclerotiorum*

The fermentation of Ag109 was carried out as previously described, standardizing for 1 × 10^9^ endospores mL^−1^. The fungus *S. sclerotiorum* was cultivated in PDA medium in a 90 mm-diameter Petri dish for five days at 25 °C. A 6 mm-diameter mycelial disc was taken from this culture and placed in the center of three Petri dishes containing BDA. Six mm-diameter wells were perforated at the two edges. In one of the wells, 200 µL of cell-free supernatant was added, while in the opposite well, 200 µL of autoclaved fermentation broth was added. Three plates inoculated only with a fungal mycelial disc were used as controls. After 96 h, the mycelial growth of the fungus was measured (in mm) with a caliper, and the percentage of inhibition was calculated.

### In vivo studies against *Sclerotina sclerotiorum* under greenhouse conditions

For this experiment, seeds of the soybean cv. Bônus were used. The seeds were disinfected by immersion in 1% (v:v) sodium hypochlorite solution for 1 min. Then, they were subjected to triple washing by immersion in sterile distilled water and dried in a safety cabinet for four hours. Subsequently, the seeds were treated with biological and chemical products, as follows:

1. Treatment 1: Control
2. Treatment 2: Certainty (active ingredient: Thiophanate + Fluazinan, applied at 100 mL/50 kg of seeds)
3. Treatment 3: Serenade (active ingredient: *Bacillus subtilis* QST 713, applied at 200 mL/50 kg of seeds)
4. Treatment 4: Ag109 (active ingredient: *Bacillus velezensis* Ag109, applied at 200 mL/50 kg of seeds). The fermentation of Ag109 was carried out as previously described, standardizing for 1 × 109 endospores mL^−1^.

Some of the control seeds and the seeds treated with biological and chemical products were inoculated with the fungus *Sclerotinia sclerotiorum*, according to Machado et al. [25]. Soybean seeds were kept for 24 h in Petri dishes containing the fungus grown in a culture medium at 25 °C. The seeds were sown in 32-cell trays (0.23 L per cell) containing sterile Carolina Soil substrate and kept in a greenhouse. One seed was sown per cell. Each tray was considered a plot, with four replications totaling 128 seeds per treatment. A completely randomized experimental design was used. Germination evaluations were performed at seven and 26 DAS. At 26 DAS, shoot and root dry mass per plant were quantified. For the experiment, the average temperature of the greenhouse was 25 ºC ± 2 ºC.

### Ag109 genomic sequencing and prediction of secondary metabolites

Ag109 was grown in LB at 150 rpm at 28 °C for 48 h for complete genome sequencing. DNA extraction was performed using a PureLinkTM Microbiome DNA Purification kit (Invitrogen, Thermo Fisher Scientific, Waltham, Massachusetts, USA). DNA integrity was verified using a 1% agarose gel and the DNA was quantified by spectrophotometry in a NanoDrop 2000/2000c (Thermo Fisher Scientific, Wilmington, Delaware, USA). Sequencing was performed on the Illumina NovaSeq 6000 platform at the Institute for Cancer Research (IPEC), Guarapuava, Paraná, Brazil.

The quality of the readings and the cutoff parameters were considered and chosen using FastQC (Andrews 2010 [26]). Then, using the Trimmomatic program [27], the raw readings were filtered based on the parameters defined by FastQC. Finally, the quality of the readings was verified after the filters to ensure that the chosen parameters were adequate.

A series of de novo assemblies were performed with different software (SPAdes and IDBA hybrid) [28], testing diverse assembly parameters. Then, the results were compared with the QUAST program [29]. The best assembly was selected based on key metrics like total alignment size, contig count, largest contig, N50 values, and gene count, as per QUAST’s reference genome. Best-assembled contigs were aligned with the *B. velezensis* NKG-1 reference genome using the Contiguator webserver to create scaffolds [30]. Gaps were filled manually with Bowtie2 for mapping and CLC Genomics Workbench 12 for gap-filling [31]. The genome’s start point was identified by comparing it with a reference strain genome, using *dnaA* as the starting gene.

The genome of Ag109 was represented circularly and compared with other reference genomes using BRIG (BLAST Ring Image Generator). To determine the species, the ANI (Average Nucleotide Identity) values were verified with other species of *Bacillus* using OrthoANI [32]. The orthoANI matrix data and information generated by the software were exported and used to create the heatmap in the R program using the ggplot2 package. The hierarchical cluster analysis used was the UPGMA.

Using different genetic databases, the Glimmer 3.02 program was used to predict genes related to plant growth promotion. The identification of possible biosynthetic gene clusters (BGCs) was performed using the antiSMASH 4.0 web server [33], which combines different genetic databases, antimicrobial molecules, and BGCs to predict the position and possible function of the clusters [34]. In addition to Ag109, the genome of *Bacillus subtilis* QST 713 and Bacillus velezensis FZB42 was analyzed by antiSMASH for further comparisons.

### Data analysis

The data from the greenhouse trials were subjected to variance analysis. When the assumptions were met, they were subjected to Tukey’s means comparison test (*p* < 0.01). Data were analyzed by the R program using the AgroR package [35].

## Results

### Nematicidal activity

By the variance analysis, a significant effect (*p* < 0.01) of treatments for the variables reproduction factor (RF) and number of nematodes per gram of roots (NGR) was observed in both experiments with *M. javanica* and *P. brachyurus*. For *M. javanica*, the treatments with Avicta, Presence, and Ag109 showed the lowest values for RF and NGR in experiment I. In experiment II, the lowest value was observed in Ag109 for RF and in Avicta, Presence, and Ag109 for NGR (Table 1). In these experiments, Ag109 allowed average reductions of 59 and 63% for RF and NGR, respectively.

**Table 1:** Effect of biological and chemical treatments for the biocontrol of *Meloidogyne javanica* and *Pratylenchus brachyurus* in soybean in vivo

Treatments^1/^	*Meloidogyne javanica*		*Pratylenchus brachyurus*	
	RF	NGR	RF	NGR
Experiment I				
Control	2.55 **a**	89.07 **a**	4.06 **ab**	36.50 **b**
Avicta	0.50 **c**	16.02 **c**	4.05 **ab**	43.48 **ab**
Votivo Prime	1.65 **b**	52.35 **b**	3.04 **b**	57.59 **a**
Presence	0.45 **c**	19.51 **c**	5.37 **a**	62.10 **a**
Ag109	0.79 **c**	25.35 **c**	1.61 **c**	20.39 **c**
Experiment II				
Control	15.78 **ab**	603.11 **a**	2.28 **a**	47.98 **b**
Avicta	9.11 **b**	315.10 **b**	2.30 **a**	60.15 **a**
Votivo Prime	21.93 **a**	732.59 **a**	2.06 **b**	63.62 **a**
Presence	13.08 **ab**	397.79 **b**	2.25 **a**	52.23 **ab**
Ag109	7.80 **c**	260.15 **b**	1.60 **b**	27.22 **b**

^1/^Means followed by the same letters in the column (per experiment) did not differ statistically by Tukey’s test (*p* < 0.01)

For *P. brachyurus*, Ag109 showed the lowest values of RF and NGR in experiment I, with 60 and 44% reductions, respectively, compared to the control. For experiment II, the lowest RF values were obtained in the treatments Ag109 and Votivo Prime, while for NGR, with Ag109. Ag109 reduced the RF and NGR by 30 and 43%, respectively, in this experiment.

### Antifungal activity

**Fig. 1:**
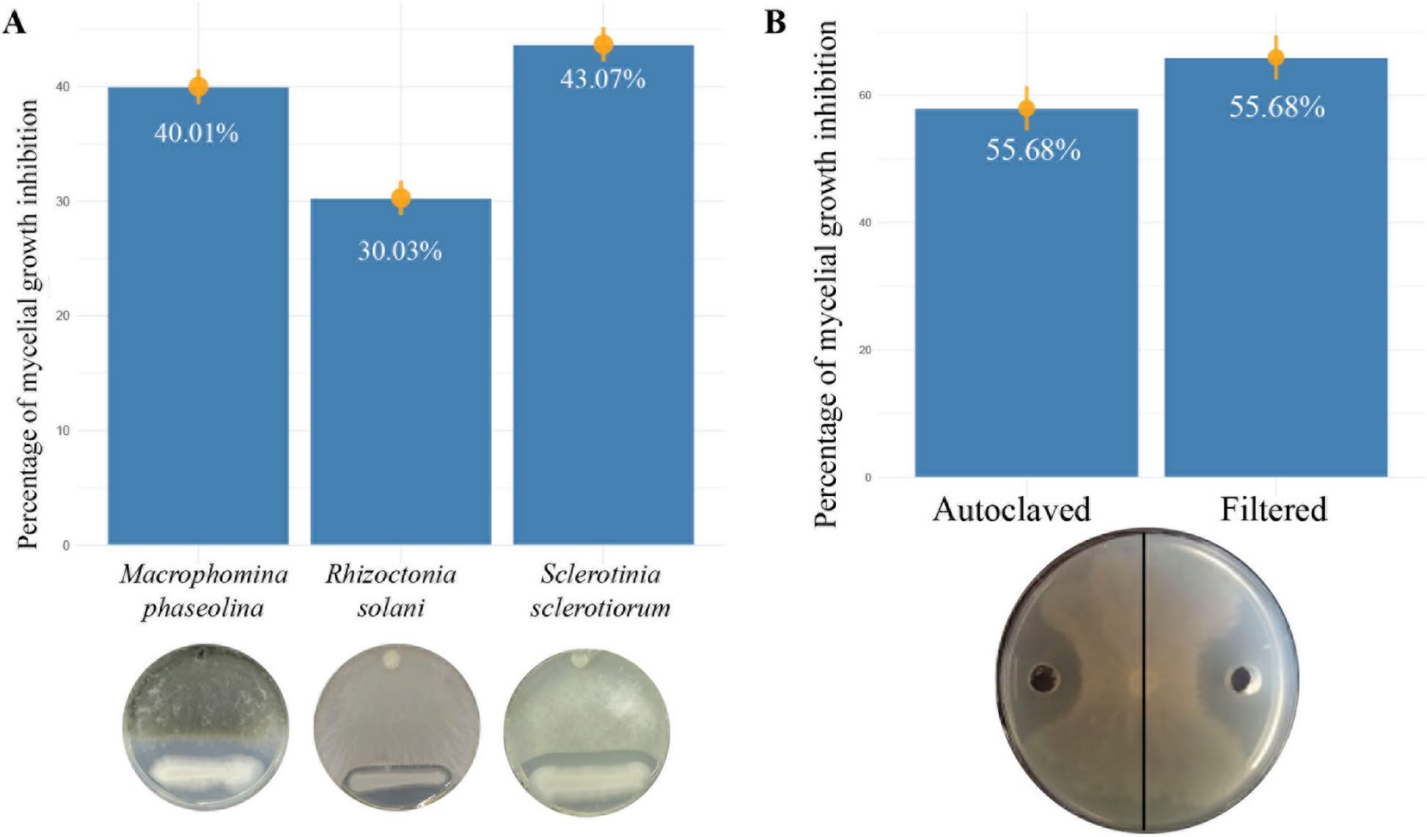
Effect of *Bacillus velezensis* strain Ag109 in relation to different phytopathogenic fungi. **A** Inhibition of mycelial growth (%) for *Macrophomina phaseolina*, *Rhizoctonia solani*, and *Sclerotinia sclerotiorum* through direct antagonism, **B** Inhibition of mycelial growth (%) of *Sclerotinia sclerotiorum* using the crude autoclaved (15 min, 120 °C) and cell-free (filtered) supernatants

The Ag109 strain showed antifungal activity against *M. phaseolina*, *R. solani*, and *S. sclerotiorum*, with mycelial growth control percentages of 40.01, 30.03, and 43.07%, respectively (Fig. 1a). No contact was observed between Ag109 and the fungi, showing that mycelial growth was paralyzed before reaching the bacterial culture and indicating antagonistic activity of the bacterial strain.

When analyzing the cell-free culture filtrate of Ag109 against *S. sclerotiorum*, a high percentage (64.10%) of growth inhibition was also observed (Fig. 1B). In addition, the autoclaved crude supernatant was also evaluated against *S. sclerotiorum* to verify the stability of the molecules. In this case, 55.9% of inhibition was detected, indicating the high control potential of the metabolites produced by Ag109.

**Fig. 2:**
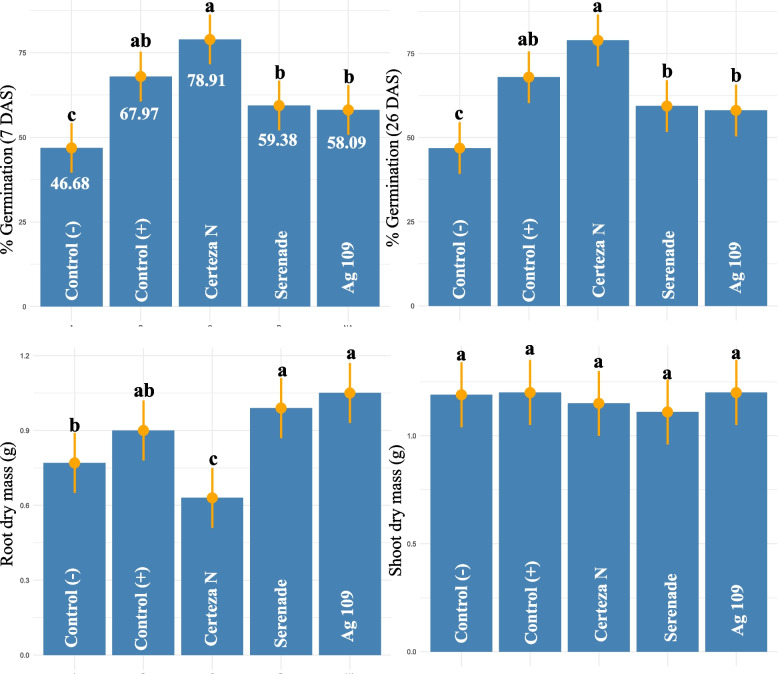
Germination percentage and root and shoot dry mass of soybean inoculated with different products and later infected with *Sclerotinia sclerotiorum*. Treatments with the same letter did not differ statistically by Tukey’s test (*p* < 0.01)

In the experiment of *S. sclerotiorum* infection in soybean seeds, a significant effect (*p* < 0.01) of treatments was observed for most traits, except for dry shoot mass. For the germination percentage of healthy plants (7 and 26 DAS), the treatments Certeza N and Control (without infection) showed the highest values, followed by Serenade and Ag109 (Fig. 2). The biological treatments showed an increase in healthy seedlings of 27 and 24%, respectively, in relation to the control (with infection), while the chemical treatment increased this trait by 68%. For root dry mass, the highest values were observed for biological treatments (Ag109 and Serenade) and the control (without infection). The chemical treatment showed the lowest value for root dry mass.

### Ag109 genome assembly and annotation

The CLC Genomics Workbench 11 and IDBA Hybrid genome assembly strategies demonstrated the best results for assembly. A BLASTN search was performed using the largest contig to find a reference genome for the CONTIGuator step. The *B. velezensis* strain Bac57 was selected to align the contigs and to generate scaffold. The scaffold contained 36 gaps that were first treated with GapCloser and then manually aligned using Bowtie2 and CLC Genomics Workbench 11. Then, the genome showed a 97.96% alignment rate of reads and a size of 3,954,988 bp. Genome annotation verified a GC content of 46.39% and 4052 CDSs, of which 24 were related to rRNA and 78 to tRNA sequences (GenBanK accession number CP099465).

### Ag109 identification and phylogenetic analysis

**Fig. 3:**
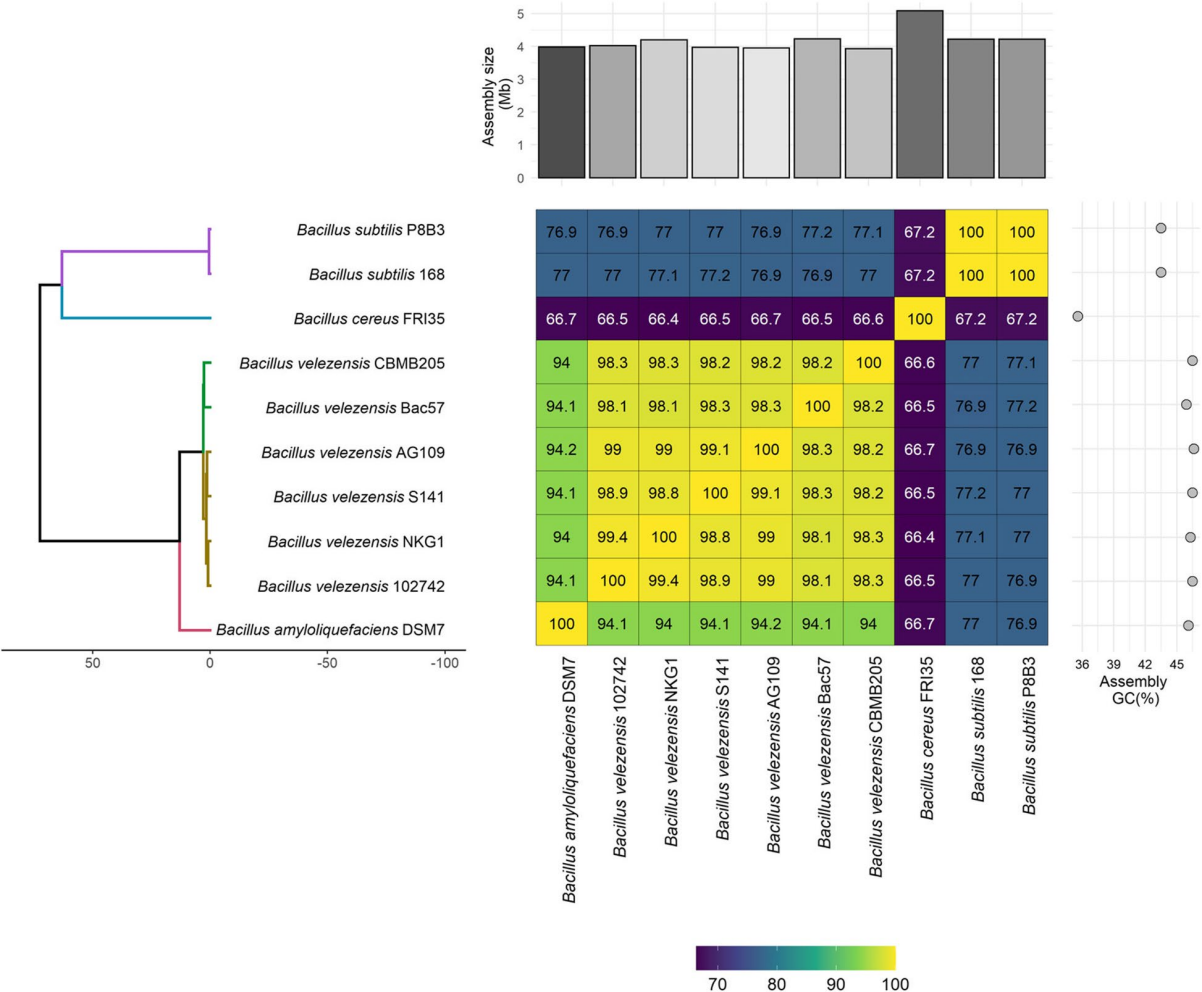
Maximum likelihood phylogenetic tree based on the 10 available genomic assemblies of *Bacillus velezensis*, *B. amyloliquefaciens*, *B. cereus*, and *B. subtilis* with heatmap annotation. Average nucleotide identity (ANI) values (%) are presented in a heatmap, ranging from lower (violet) to higher sequence identity (green-yellow), and clustered according to the phylogenetic tree. The heatmap was annotated with a bar chart showing the varying sizes (Mb) of all 10 assemblies (at the top) and their respective GC (%) content (right-hand side)

**Fig. 4:**
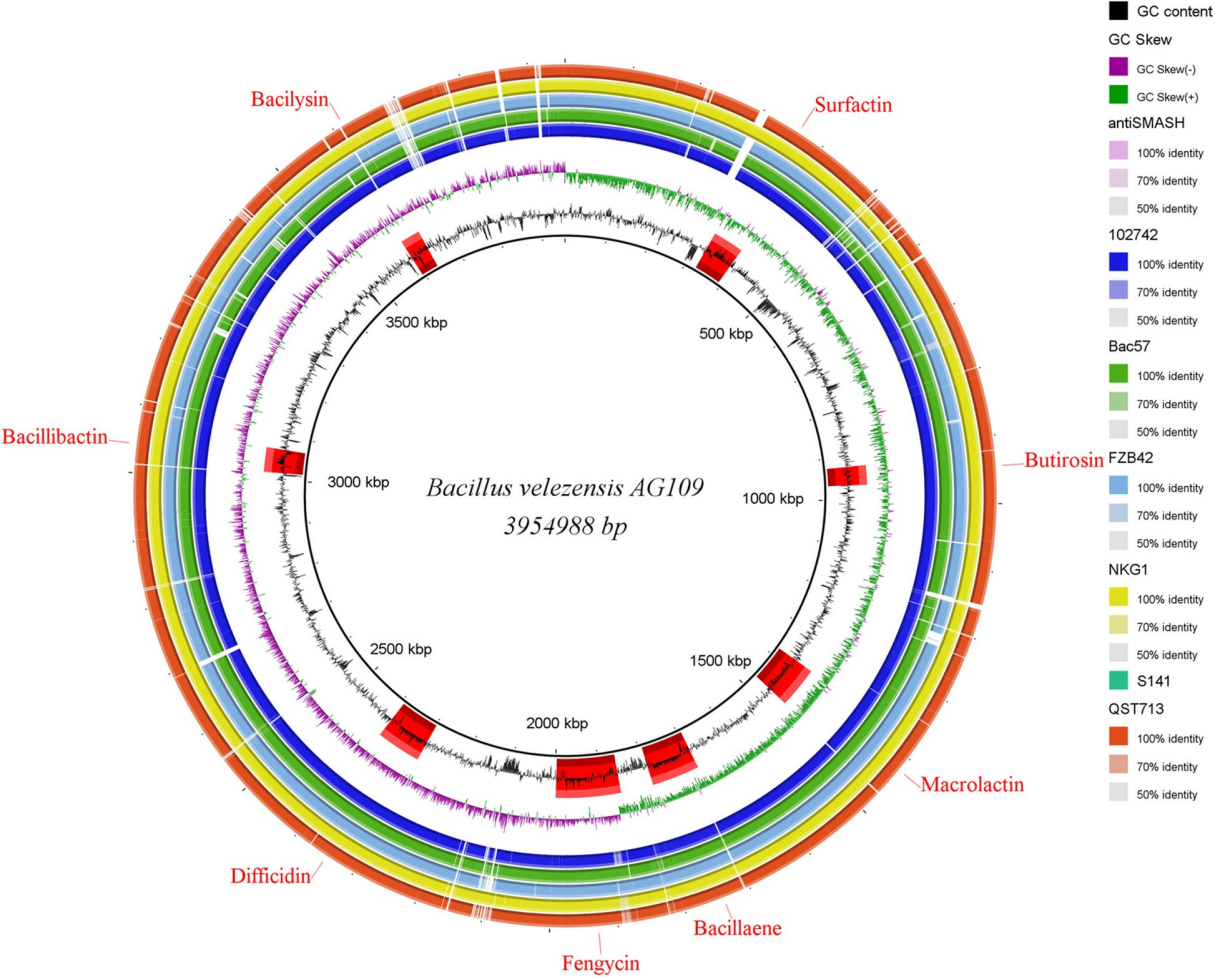
Circular representation of the genome of *Bacillus velezensis* Ag109 using the program BRIG. From the inside to the outside, the legends are as follows: GC content, GC skew, BGC position on the genome indicated by antiSMASH: 102742, BAC 57, FZB42, NKG1, and QST 713, respectively

Comparing Ag109 with the main species of the *B. subtilis* group, it was observed that the mean nucleotide identity (ANI) and digital DNA-DNA hybridization (dDDH) were superior in the groups of *B. velezensis* isolates, with values ranging from 98.3 to 99.9% for ANI and 84.3 to 92.4% for dDDH (Table S1; Fig. 3). Compared with *B. amyloliquefaciens* (DSM7 and MT 45 strains), the ANI and dDDH values were 94.0 – 94.1% and 55.7%, respectively, while for *B. subtilis* (168 and D125 strains), they were 77.2 – 77.3% and 20.7%, respectively. The values for *B. cereus* (FR135 strain) were 66.3 and 38.9%, respectively. In the comparison performed with Gegenees and orthoANI/GGDC, it was observed that Ag109 is located within the cluster containing most of *B. velezensis* (Fig. 3). The circular genome of Ag109 is represented in Fig. 4.

### Genetic basis for plant growth-promoting activity

The Ag109 genome has several genes that encode proteins associated with plant growth-promoting activity (Table 2). For instance, ten putative genes (*trpA, trpB, trpC, trpD, trpE, trpF, ywdH, ysnE, ywkB,* and *amhX*) involved in the production of indole-3-acetic acid were identified. In addition, other genes related to biofilm formation, volatile compounds, glucose dehydrogenase, phenazine, trehalose, heat and cold shock, glycine-betaine, and peroxidases were identified.

**Table 2:** Genes detected in the *Bacillus velezensis* Ag109 genome predicated to be involved in plant growth-promoting activity

Gene ID	Gene name	Protein coded by the gene
Genes detected in *Bacillus velezensis* Ag109 genome predicated to be involved in the production of indole acetic acid (IAA)		
AG109_002169	*trpA*	Tryptophan synthase subunit alpha
AG109_002170	*trpB*	Tryptophan synthase subunit beta
AG109_002172	*trpC*	Indole-3-glycerol phosphate synthase
AG109_002173	*trpD*	Anthranilate phosphoribosyl transferase
AG109_002174	*trpE*	Anthranilate synthase component I
AG109_002171	*trpF*	Phosphoribosyl anthranilate isomerase
AG109_002794	*ywdH*	Aldehyde dehydrogenase
AG109_002277	*ysnE*	GNAT family N-acetyltransferase
AG109_003575	*ywkB*	Auxin efflux carrier family protein
AG109_000283	*amhX*	Amidohydrolase
Genes detected in *Bacillus velezensis* Ag109 genome predicated to be involved in the production of spermidine and polyamine		
AG109_001869	*bioI*	Biotin biosynthesis cytochrome P450
AG109_003621	*speE*	Spermidine synthase
AG109_000568	*bltD*	Spermidine acetyltransferas
Genes detected in *Bacillus velezensis* Ag109 genome predicated to be involved in the production of volatile compound (VOC)		
AG109_003462	*budA*	Acetolactate decarboxylase
AG109_002658	*ilvB*	Acetolactate synthase
AG109_000385	*yczG*	Transcriptional regulator
AG109_000626	*bdhA*	2,3-butanediol dehydrogenase
AG109_000792	*acoA*	Acetoin dehydrogenase
Genes detected in *Bacillus velezensis* Ag109 genome predicated to be involved in biofilm formation, development, and regulation		
AG109_001508	*ylbF*	Controls biofilm development
AG109_001713	*ymcA*	Biofilm development
AG109_003197	*ioIW*	Scyllo-inositol 2-dehydrogenase (NADP(1) involved in biofilm formation protein
AG109_001658	*sigD*	RNA polymerase sigma factor for flagellar operon and Biofilm formation
AG109_002394	*sinR*	Master regulator of biofilm formation
AG109_002902	*luxS*	S-ribosyl homocysteine lyase for Quorum sensing Biofilm formation
AG109_003279	*rpoN*	RNA polymerase sigma-54 factor for Biofilm formation
AG109_003392	*csrA*	Carbon storage regulator for Biofilm formation
AG109_003398	*flgM*	Negative regulator of flagellin synthesis f*lgmfor* Biofilm formation
AG109_003421	*wecB*	UDP-N-acetylglucosamine 2-epimerase for Biofilm formation
Genes detected in *Bacillus velezensis* Ag109 genome predicated to be related to Glucose dehydrogenase		
AG109_000267	*ycdF*	Glucose 1-dehydrogenase
AG109_000391	*gdh*	Glucose 1-dehydrogenase
AG109_002312	*zwf*	Glucose-6-phosphate dehydrogenase
AG109_003413	*tuaD*	UDP-glucose 6-dehydrogenase
Genes detected in *Bacillus velezensis* Ag109 genome predicated to be involved in Phenazine production and Trehalose metabolism		
AG109_000819	*phzF*	PhzF family phenazine biosynthesis isomerase
AG109_000756	*treP*	PTS system trehalose-specific EIIBC component
AG109_000758	*treR*	Trehalose operon repressor
Genes detected in *Bacillus velezensis* Ag109 genome predicated to be involved in heat and cold shock		
AG109_000061	*hslR*	Ribosomal RNA binding protein involved in 50S recycling heat shock protein
AG109_000608	*GroeL*	Heat shock protein 60 kDa family chaperone GroEL
AG109_000607	*GroeS*	Heat shock protein 10 kDa family chaperone GroES
AG109_000503	*cspC*	Cold shock protein CspC
AG109_000891	*cspB*	Cold shock-like protein CspB
AG109_002087	*cspD*	Cold-shock protein CspD
Genes detected in *Bacillus velezensis* Ag109 genome predicated to be involved in Glycine-betaine production		
AG109_000280	*opuAA*	Glycine/proline betaine ABC transporter ATP-binding protein OpuAA
AG109_000281	*opuAB*	Glycine/proline betaine ABC transporter permease subunit OpuAB
AG109_000282	*opuAC*	Glycine/betaine ABC transporter
AG109_002849	*opuD*	Glycine betaine transporter OpuD
Genes detected in *Bacillus velezensis* Ag109 genome predicated to be related to Peroxidases		
AG109_002083	*bsaA*	Glutathione peroxidase
AG109_000848	*bcp*	Thiol peroxidase

### AntiSMASH analysis of secondary metabolites

**Fig. 5:**
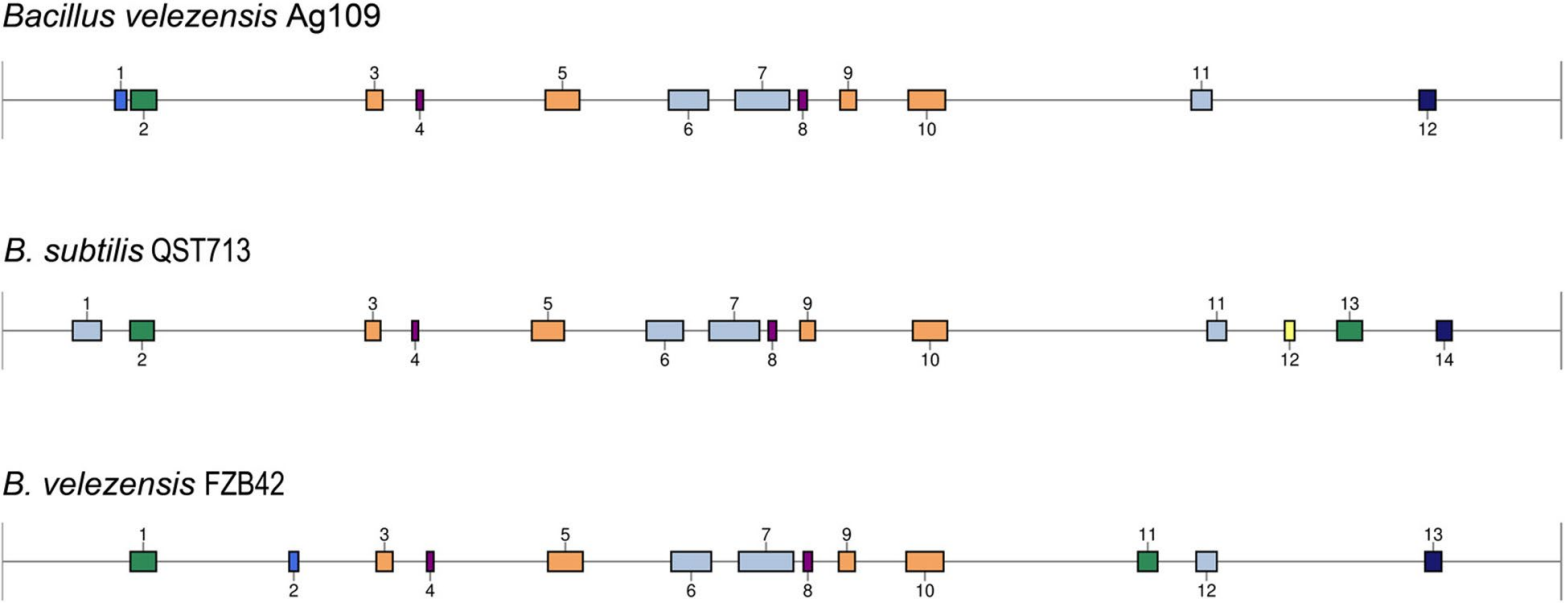
Comparison of gene clusters between *Bacillus velezensis* Ag109 with *Bacillus subtilis* QST 713 and *Bacillus velezensis* FZB42. Cluster no.12 in QST 713 genome not presented in the other genome

Using the antiSMASH 5.1.0 webserver, 13 biosynthetic gene clusters (BGCs) were identified in the genome of Ag109 (Table 3). Six BGCs showed 100% similarity and are linked to the synthesis of bacilisin, bacilibactin, difficidin, fengicin, bacilaene, and macrolactin. One cluster showed 56% similarity with surfactin synthesis, and another, 7% similarity with the synthesis of butyrosine A and B. Four clusters showed no similarity with the database. The 13 gene clusters identified in Ag109 were also present in the commercial strains QST 713 and FZB42 (Fig. 5).

**Table 3:** Biosynthetic gene clusters (BGCs) found within the *Bacillus velezensis* Ag109 genome using the webserver antiSMASH 5.1.0 and comparison with strains QST713 and FZB42

Ag 109		Gene cluster location				Presence ( +) or absence (-)	
**Cluster no**	**Type**^**a**^	**From**	**To**	**Compound**	**Size (nt)**	**QST713**	**FZB42**
1	thiopeptide, LAP	286448	316184	unknown	29736	+	-
2	NRPS	327006	392089	Surfactin	65083	+	+
3	PKS-like	924352	965596	Butirosin A/ B	41244	+	+
4	terpene	1051222	1068393	unknown	17171	+	+
5	transAT-PKS	1378445	1464831	macrolactin H	86386	+	+
6	transAT-PKS, T3PKS, NRPS	1690964	1791515	bacillaene	100551	+	+
7	NRPS, transAT_PKS, betalactone	1859818	1997193	fengycin	137375	+	+
8	terpene	2020230	2042113	unknown	21883	+	+
9	T3PKS	2125784	2166,884	unknown	41100	+	+
10	transAT-PKS	2298784	2392583	difficidin	93799	+	+
11	NRP-metallophore, NRPS, RiPP-like	3016556	3068,347	bacillibactin	51791	+	+
12	other	3594830	3636248	bacilysin	41418	+	+

^a^*NRPS* non-ribosomal peptide synthetase, *NRP* non-ribosomal peptide, *PKS* polyketide synthetase, *AT* acetyltransferase, *T3PKS* type 3 Pks

## Discussion

Plant diseases caused by soil-transmitted pathogens are considered one of the leading causes of yield loss in various crops [14, 36]. Consequently, a variety of control strategies have been suggested. Among them, biocontrol agents are an essential strategy for controlling soil pathogens. These agents can act through parasitism, antibiosis, competition for the host and nutrients, and induction of plant resistance [37]. *Bacillus* is considered an important biocontrol agent and has been widely used against several pathogens in different crops [18, 38–40]. The current study identified a bacterial strain, Ag109, from maize rhizospheric soil that exhibits a strong antagonistic effect against *S. sclerotiorum*. This strain was further investigated to evaluate its potential as a biocontrol, for *M. javanica* and *P. brachyurus*, and Ag109 showed significant suppression. These nematode species are considered a crucial obstacle to soybean cultivation in Brazil. *P. brachyurus* is a migratory endoparasite that causes lesions on the root cortex, leading to root deterioration. This pathogen is highly polyphagous and commonly found in tropical regions, mainly in soybean-corn crop succession systems under no-tillage [41]. Likewise, many root-knot nematodes, mainly *M. javanica* and *M. incognita*, can be found in practically all soybean production fields. The parasitism caused by this nematode leads to hypertrophy and hyperplasia of root cells, compromising plant development [5].

Furthermore, Ag109 showed a significant suppression effect against the primary soil fungi in soybean. For *S. sclerotiorum*, this strain increased the number of healthy seedlings and root dry mass relative to the control, indicating the potential for promoting root growth. Several studies have indicated the multifunctional potential of some *Bacillus* species/strains for plant protection against phytopathogenic microorganisms and their growth-promoting action on plants [42–45]. *Bacillus* can improve soil nutrient availability through improved nitrogen supply, phosphate and potassium solubilization, and siderophore production. In addition, these bacteria secrete hormones and volatile organic compounds associated with root development, improving plant nutrient uptake. Associated with these characteristics, *Bacillus* can improve plant resistance against soil pathogens through competition for niches and nutrients, production of antagonist compounds, and induction of systemic plant resistance [44, 45].

By genomic analysis, Ag109 was identified as *B. velezensis* and clusters of specific genes related to secondary metabolite biosynthesis and root system promotion were identified. *B. velezensis* is an important biocontrol agent and plant growth promoter in several crops of economic importance [17, 22, 45–49]. For instance, the FZB42 isolate, which has already been published in over 140 articles, has been related to the identification of antimicrobial compounds responsible for biocontrol, the expression of genes involved in plant-bacteria interactions, the study of small regulatory RNAs (sRNAs), and the modification of enzymes involved in the synthesis of antimicrobial compounds. This information is deposited in the ‘AmyloWiki’ database (http://amylowiki.top/) [22]. The QST 713 strain, present in a commercial product, which performed well in the tests, share the same clusters as Ag109 with exception for a single cluster present only in QST 713.

In the Ag109 strain, lipopeptides (LPs) (surfactin and fengycin) were identified, which have an essential effect on pathogen suppression, stimulation of plant defense mechanisms, and biofilm formation – a key factor for the successful colonization of biological control agents [50–52]. Its mode of action in antifungal activity is related to its amphiphilic nature and its interaction with the target cell membrane, resulting in changes in structure and permeability [51–53]. In this context, this class of metabolites may be directly related to the antifungal activity of Ag109 in vitro and in vivo assays against phytopathogenic fungi. It may be the target of future in silico and downstream studies. For nematodes, Kavitha et al. [54] found that the LPs surfactin and iturin produced by *B. subtilis* were responsible for suppressing the hatching of *M. incognita* eggs and played an essential role in juvenile mortality.

Another large class of secondary metabolites identified in Ag109 were polyketides (PKS) (difficidin, bacillaene, and macrolactin), which play an essential role in antimicrobial activity, acting in the selective inhibition of protein synthesis [55, 56]. Chen et al. [57] found that difficidin produced by the FZB42 isolate had a significant antagonistic effect on *Erwinia amylovora*, which causes fire blight in fruit trees. In turn, Yuan et al. [58] demonstrated that the macrolactin produced by the NJN-6 isolate (*B. amyloliquefaciens*) affects the control of *Ralstonia solanacearum*, an important soil pathogen. In this context, the Ag109 strain can potentially control soil bacterial diseases that have not yet been explored.

From the 13 clusters identified in the Ag109 genome, four had no similarity to the database, so their products still need to be identified and described, opening opportunities for further studies. Likewise, Teixeira et al. [17] mined the genome of *B. velezensis* CMRP 4490 and failed to identify five of the 13 clusters of secondary metabolites, indicating new clusters of these metabolites.

In addition to producing antimicrobial metabolites, the Ag109 genome has genes related to plant growth-promoting activity. Several genes identified are functionally linked to auxin synthesis and play important roles in the stimulation of plant development [59, 60]. In addition, genes related to spermidine and polyamine production were found, which are suggested to have a role in plant development and growth promotion, involving the production of active metabolites such as steroids, vitamin D3, cholesterol, cytokinin, statins, and terpenes [60]. This strain also has biofilm formation, development, and regulatory genes. Bacterial biofilms are a collective way of life that confer emergent properties on the inhabitants of these communities, influencing plant growth and behavior and directly protecting against soil pathogens through the secretion of compounds [61]. Thus, the Ag109 strain has excellent potential for developing a multifunctional microbial inoculant that combines the ability to biocontrol various soil pathogens (fungi and nematodes) and promote soybean growth.

## Conclusion

Ag109 strain shows considerable promise as the basis for a multifunctional microbial inoculant designed for soybeans. This inoculant would offer biological control and enhance plant growth, with particular efficacy in suppressing nematodes like *M. javanica* and *P. brachyurus*. Future studies should explore the mechanisms behind Ag109’s antagonistic effects and growth-promotion properties and its performance in field conditions against a broader spectrum of pathogens and under varying environmental conditions. This would pave the way for developing more effective, environmentally friendly plant protection and enhancement strategies, contributing significantly to the sustainability and productivity of soybean cultivation.

### Supplementary Information

**Supplementary Material 1.**

## Data Availability

The datasets used and/or analyzed during the current study are available from the corresponding author on reasonable request.
