# Supplementary material for: Complete genome sequence of Bacillus velezensis strain Ag109, a biocontrol agent against plant-parasitic nematodes and Sclerotinia sclerotiorum
Source: BMC Microbiol. 2024 Jun 7;24:194. doi: 10.1186/s12866-024-03282-9 (PMC11157790; doi:10.1186/s12866-024-03282-9)
Supplement: Supplementary file 1 — Supplementary Material 1. [file 12866_2024_3282_MOESM1_ESM.docx]

Supplementary table for

**Complete genome sequence of *Bacillus velezensis* strain Ag 109, a biocontrol agent for suppression of soilborne pathogens of soybean**

**BMC Microbiology**

Silas Mian^1^, Andressa Cristina Zamboni Machado^2^, Rodrigo Thibes Hoshino^1^, Mirela Mosela^3^, Allan Yukio Higashi^1^, Gabriel Danilo Shimizu^1^, Gustavo Manoel Teixeira^3^, Alison Fernando Nogueira^1^, Renata Mussoi Giacomin^4^, Luriam Aparecida Brandão Ribeiro^2^, Alessandra Koltun^5^, Rafael de Assis^1^, and Leandro Simões Azeredo Gonçalves^1*^

*^1^Agronomy Department, Universidade Estadual de Londrina, Londrina, 86097-570, Paraná, Brazil.*

*^2^Nematology Department, Instituto Agronômico do Paraná, Londrina, 86047-902, Paraná, Brazil.*

*^1^Microbiology Department, Universidade Estadual de Londrina, Londrina, 86097-570, Paraná, Brazil.*

*^4^Biology Department, Universidade Estadual do Centro Oeste, Guarapuava, 85015-430, Paraná, Brazil.*

*^5^Center for Molecular Biology and Genetic Enginnering, UNICAMP, Campinas, 13083-875, São Paulo, Brazil.*

* Corresponding author: E-mail: leandrosag@uel.br ORCID: 0000-0001-9700-9375

**This document includes:**

**Supplementary Table –**

***Supplementary Table S1** Genome comparison of Ag109 with other *Bacillus* species.

**Table S1** Genome comparison of Ag109 with other *Bacillus* species.

| Strains | Accession n^o^ GenBank | dDDH(%)^1/^ | ANI (%) | GC (%) | Bp (Gp) | Source |
| --- | --- | --- | --- | --- | --- | --- |
| ***Bacillus velezensis*** | | | | | | |
| CMLL6 |  |  |  | 46,48 | 3,98 |  |
| QST713 | CP025079 | 88,1 | 98,51 | 45,9 | 4,23 | Commercial product Bayer (Pandin et al, 2010) |
| FZB42 | CP000560.2 | 91,0 | 98,93 | 46,5 | 3,92 | Soil (Chen et al., 2009) |
| CBMB205 | CP011937.1 | 84,3 | 98,31 | 46,5 | 3,93 | Unpublished data (Lee and Jeon, 2015) |
| Bac57. | CP033054.1 | 84,9 | 98,31 | 45,89 | 4,23 | Red Sea lagoon |
| NKG1 | CP024203.1 | 91,1 | 98,92 | 46,3 | 4,2 | Volcanic soils at the Changbai mountains (Ge et al., 2016) |
| S141 | AP018402.1 | 92,4 | 99,11 | 46,5 | 3,97 | Soybean rhizosphere (Sibponkrung et al., 2017) |
| LABIM40 | CP025079.1 | 55,7 | 99,97 | 46,5 | 3,97 | Contaminant (Baptista et al., 2018) |
| **Bacillus *amyloliquefaciens*** | | | | | | |
| DSM7 | NC_014551.1 | 55,7 | 94,05 | 46,1 | 3,98 | Wheat Rhizosphere (Hao et al., 2012) |
| MT45 | NC_014551.1 | 55,7 | 94,12 | 46,1 | 3,89 | Not specified |
| ***Bacillus subtilis*** | | | | | | |
| D125 | CP020102.1 | 20,7 | 77,26 | 43,6 | 4,14 | Soil (Niazi et al., 2014) |
| ***Bacillus cereus*** | | | | | | |
| FR135 | CP003747.1 | 38,9 | 66,30 | 35,4 | 5,48 | Not specified |

^1/^dDDH: digital DNA-DNA hybridization, ANI: average nucleotide identity, GC: contente of guanine and cytosine on the genome, and Bp: genome size in gigabases (10^6^).
